# Greater thermoregulatory strain in the morning than late afternoon during judo training in the heat of summer

**DOI:** 10.1371/journal.pone.0242916

**Published:** 2020-12-01

**Authors:** Hidenori Otani, Takayuki Goto, Yuki Kobayashi, Minayuki Shirato, Heita Goto, Yuri Hosokawa, Ken Tokizawa, Mitsuharu Kaya

**Affiliations:** 1 Faculty of Health Care Sciences, Himeji Dokkyo University, Himeji, Hyogo, Japan; 2 National Institute of Technology, Akashi College, Akashi, Hyogo, Japan; 3 Meiji Gakuin University, Tokyo, Japan; 4 Kyushu Kyoritsu University, Kitakyushu, Fukuoka, Japan; 5 Faculty of Sport Sciences, Waseda University, Tokorozawa, Saitama, Japan; 6 National Institute of Occupational Safety and Health, Kiyose, Tokyo, Japan; 7 Hyogo University of Health Sciences, Kobe, Hyogo, Japan; West Virginia University, UNITED STATES

## Abstract

**Purpose:**

The time-of-day variations in environmental heat stress have been known to affect thermoregulatory responses and the risk of exertional heat-related illness during outdoor exercise in the heat. However, such effect and risk are still needed to be examined during indoor sports/exercises. The current study investigated the diurnal relationships between thermoregulatory strain and environmental heat stress during regular judo training in a judo training facility without air conditioning on a clear day in the heat of summer.

**Methods:**

Eight male high school judokas completed two 2.5-h indoor judo training sessions. The sessions were commenced at 09:00 h (AM) and 16:00 h (PM) on separate days.

**Results:**

During the sessions, indoor and outdoor heat stress progressively increased in AM but decreased in PM, and indoor heat stress was less in AM than PM (mean ambient temperature: AM 32.7±0.4°C; PM 34.4±1.0°C, *P*<0.01). Mean skin temperature was higher in AM than PM (*P*<0.05), despite greater dry and evaporative heat losses in AM than PM (*P*<0.001). Infrared tympanic temperature, heart rate and thermal sensation demonstrated a trial by time interaction (*P*<0.001) with no differences at any time point between trials, showing relatively higher responses in these variables in PM compared to AM during the early stages of training and in AM compared to PM during the later stages of training. There were no differences between trials in body mass loss and rating of perceived exertion.

**Conclusions:**

This study indicates a greater thermoregulatory strain in the morning from 09:00 h than the late afternoon from 16:00 h during 2.5-h regular judo training in no air conditioning facility on a clear day in the heat of summer. This observation is associated with a progressive increase in indoor and outdoor heat stress in the morning, despite a less indoor heat stress in the morning than the afternoon.

## Introduction

A greater thermoregulatory strain (i.e. higher body temperature and heart rate [HR]) has been reported in the morning exercise session from 09:00 h than in the late afternoon exercise session from 16:00 h in high school athletes during 3-h moderate-intensity baseball training [1] and 2-h high-intensity football training [2] in the heat outdoors under a clear sky. Given that there were no time-of-day differences in ambient temperature (T_a_) and wet-bulb globe temperature (WBGT) between the sessions, these observations occurred because of the differences in environmental heat stress during exercise which increased with rising solar radiation and elevation angle in the morning but decreased with falling solar radiation and elevation angle in the late afternoon [1, 2]. Otani and colleagues [1, 2] therefore concluded that 2–3 h moderate- to high-intensity exercise in the heat of summer under a clear sky may be at a relatively higher risk for developing exertional heat-related illness in the morning from 09:00 h than in the late afternoon from 16:00 h. Those conclusions indicate that the diurnal variations in environmental heat stress affect thermoregulatory responses and the risk of exertional heat-related illness during outdoor exercise in a hot environment. However, such effect and risk are still needed to be examined during indoor sports/exercises.

Judo is a popular combat sport in junior high school and high school athletics in Japan. Judo has been reported to have one of highest numbers of exertional heat-related illness among school organized sports activities in Japan [3]. This is possibly due to a luck of air conditioning in the most judo facilities owing to its high running costs. Hence, majority of judo training sessions during the summer are performed under severe heat stress conditions, including high T_a_ and relative humidity (RH). This means that an increase in outdoor heat stress raises indoor heat stress in a judo facility in the heat of summer. Sport-specific characteristics of judo may also be responsible for increasing the risk of heat-related illnesses, since judo has high-density efforts and is a high-intensity sport [4] that requires a high-level of strength and endurance performance [5]. Moreover, there are seven weight divisions in judo and athletes are generally required to lose weight before competitions which may induce cumulative dehydration or hypohydration. Also, heavier weight class judokas have high body mass index which produces more metabolic heat and is less efficient in dissipating heat during exercise [6]. Both dehydration/hypohydration and high body mass index have been recognised as a thermoregulatory challenge and a higher risk for developing exertional heat-related illness during exercise in the heat [6]. To our knowledge, only one study has reported the impact of regular judo training on physiological responses in no air conditioning facility in the summer [7]. However, the study [7] was limited to the assessment of hydration status during 90 min regular judo training under a moderate heat stress (29.5°C T_a_). Therefore, no study has investigated the effect of time-of-day changes in indoor and outdoor heat stresses on thermoregulatory responses during regular judo training in a facility without air conditioning in the heat of summer.

The aim of the current study was therefore to investigate the diurnal relationships between thermoregulatory responses and indoor and outdoor heat stresses during regular judo training in a judo training facility without air conditioning in the heat of summer. We hypothesised that thermoregulatory strain during the training would be greater in the morning than in the late afternoon due to a progressive increase in heat stress in both indoor and outdoor environments during the morning.

## Methods

### Participants

Participants were eight healthy, heat-acclimatized males who belonged to a high school judo team (mean±standard deviation [SD]; age 16.5±1.0 y, height 167±6 cm, body mass 66±11 kg, BMI 23±4 kg·m^−2^, years of training 6±2 y). All data collections were completed in August to ensure that participants were naturally acclimated to the heat. Their weight divisions were 2 extra lightweight, 1 half lightweight, 2 lightweight, 2 half middleweight and 1 middleweight. They trained ~5 days per week and performed a similar protocol of training in the current study more than 12 weeks. All participants and their parents received written information regarding the nature and purpose of this study prior to participation in the study. Following an opportunity to ask questions, a written statement of consent was signed by their parents. The protocol employed was approved by the local Ethics Advisory Committee of Himeji Dokkyo University (REF: 19–05) and was conducted in accordance with principles of the Declaration of Helsinki.

### Experimental protocol

All participants completed two 2.5-h regular judo training sessions in a judo facility. A building was located approximately 15 m from the north side of the facility; however, there were no obstructions to shield the sun within a 50 m radius from the east, south and west sides of the judo facility. The judo facility was a one-story building with a floor space of 225 m^2^ (15 m × 15m). The judo facility had no air conditioning and there were windows on east and west sides of the wall, which were kept open during the sessions. The sessions were commenced at two different times-of-day: 09:00 h (AM) and 16:00 h (PM). The present study was conducted in early-August on a completely clear day, and PM trial was conducted first and AM trial was carried out two days later. A normal training session took place two days before the first trial (PM trial) but no exercise was permitted during the 24 h prior to the trials. Participants were dressed in the same judo uniform (jacket, pants, belt) in both trials. In the current study, participants wore a T-shirt under the judo uniform to protect surface skin temperature thermistor probes at the chest and upper arm during the sessions. This judo ensemble was 2.5±0.1 kg of total weight. There were no studies reporting the intrinsic clothing insulation (*R*_cl_), the clothing area factor (*f*_cl_) and the evaporative resistance of clothing (*R*_e,cl_) of judo uniform. The present study therefore used the similar clothing to estimate *R*_cl_, including 0.18 clo of short sleeve, sport shirt, 0.50 clo of double-breasted suit, jacket (denim), and 0.32 clo of straight, long, loose (denim) [8]. A clo is a unit of thermal insulation for clothing: one clo can be defined as the amount of insulation that allows the transfer of 1 W·m^−2^ with a temperature gradient of 0.155°C between two surfaces (0.18°C·m^2^·h·kcal^−1^). Total *R*_cl_ was calculated as 0.77 × the sum of these *R*_cl_ [8]. The *f*_cl_ was calculated as (0.305 × total *R*_cl_) + 1.0 [8]. As a result, total *R*_cl_ was 0.770 clo or 0.119 W·(m^2^·°C)^−1^ and *f*_cl_ was 1.23. Since this total *R*_cl_ was similar to that of baseball uniform-temperate weather (0.762 clo) and football uniform-warm weather (0.795 clo) [9], the current study used *R*_e,cl_ of these uniforms which were 0.022 W·(m^2^·kPa)^−1^.

Participants entered a laboratory which was close to the judo facility after a 2 h fast in each trial with the exception of plain water, which was allowed until 30 min before the start of the trials. Upon arrival, participants first emptied their bladder and thereafter nude body mass was measured to the nearest 10 g (AD6205B, A&D Co., Ltd., Tokyo, Japan). Surface skin temperature thermistor probes (ITP082-25, Nikkiso-Therm Co., Ltd., Musashino, Tokyo, Japan) were attached to four sites (chest, upper arm, thigh and calf) under the clothing without preventing range of motion. A weighted average of chest (0.3), upper arm (0.3), thigh (0.2) and calf (0.2) skin temperatures was used to calculate mean skin temperature (T_sk_) [10]. Gastrointestinal thermometry has been shown to be a valuable device for core temperature (T_core_) assessment in the field and athletics settings [11]. However, the current study measured an infrared tympanic temperature (T_ty_) to estimate T_core_ due to the restriction from pharmaceutical affairs law in Japan using gastrointestinal thermometry. T_ty_ was measured using an infrared tympanic thermometer (GeniusTM 2, Covidien, Mansfield, MA, USA). In each measurement, two consecutive readings were obtained. All T_ty_ measurements were taken by a single operator, using the recommended technique [12]. To avoid the increased effects of increasing T_a_ on T_ty_ in the heat [13], the thermometer was stored inside a cooling box during the trial. The temperature inside this box was maintained by ice packs at about 25°C. Thermal sensation (TS) was measured using a 9-point scale [14]. All pre-exercise measurements were carried out in the laboratory in a temperate environment (25–27°C T_a_) because prior heat stress exposure may increase thermoregulatory strain during subsequent exercise-heat stress in the morning than in the afternoon [15].

**Fig 1:**
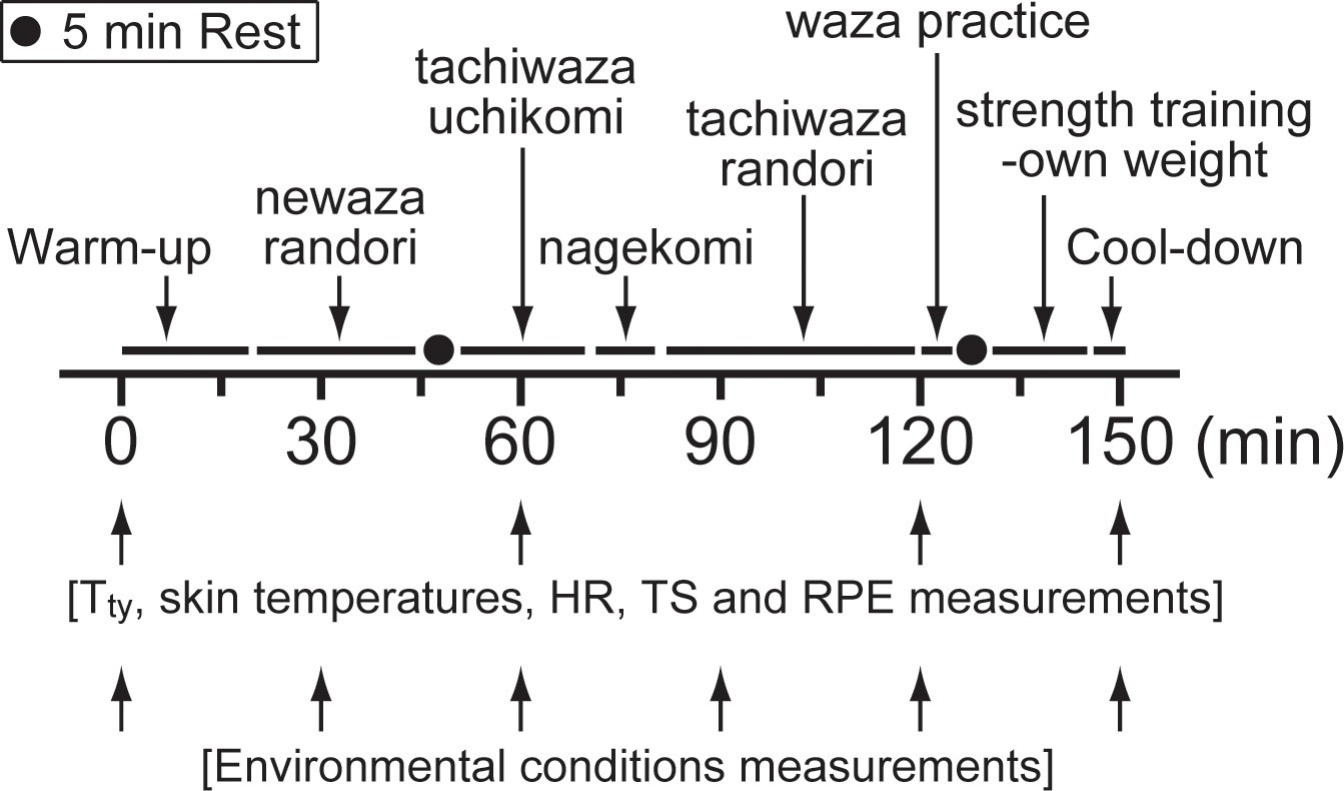
Schematic representation of the experimental protocol. T_ty_, infrared tympanic temperature; HR, heart rate; TS, thermal sensation; RPE, rating of perceived exertion.

Participants then entered the judo facility and commenced a 2.5-h training session. Participants started the sessions in a dry judo uniform. Participants received airflow during the sessions which was directed from 3 corners to the centre of the facility by 3 industrial fans, using a 0.5 m blade diameter fan (SF-50FS-1VP, Suiden Co. Ltd., Sangocho, Osaka, Japan), to prevent high levels of hyperthermia during exercise in the heat [16]. Mean air velocity in the judo facility was about 2.5 km·h^−1^. Both training sessions were led by the same judo instructor to retain consistency between the two experiments. The content of training session in both trials was as follows: warm-up (20 min: running, dynamic-stretching and ukemi); newaza randori (25 min); rest (5 min); tachiwaza uchikomi (20 min); nagekomi (10 min); tachiwaza randori (40 min); waza practice (5 min); rest (5 min); strength training-own weight (15 min); and cool-down (5 min: static-stretching) (Fig 1). During the sessions, T_ty_ and TS were assessed every 60 min and at the end of the training (Fig 1). To determine whole-body perception of effort, rating of perceived exertion (RPE) was assessed every 60 min and at the end of the training using the 6–20 RPE scale [17] (Fig 1). Skin temperatures (thermometer N543R, Nikkiso-Therm Co., Ltd., Musashino, Tokyo, Japan) and HR (HR monitor A370, Polar Electro, Kempele, Finland) were also recorded every 60 min and at the end of the training (Fig 1). Participants were free to ingest plain water maintained at about 30°C during the sessions. Following the sessions, participants returned to the laboratory, removed the probes and re-measured nude body mass to allow the estimation of total sweat loss.

### Environmental measurements

Environmental conditions were measured at both inside and outside of the judo facility. Indoor environmental conditions were measured 1.5 m above the floor. Outdoor environmental conditions were measured 1.5 m above a dark asphalt pavement close to the judo facility. T_a_, RH, black globe temperature (T_g_), and WBGT were measured using a WBGT meter (WBGT-203A; Kyoto Electronics Industry Co., Ltd., Fukuchiyama, Kyoto, Japan) every 30 min. Air velocity was measured using an anemometer (AM-4214SD; Mother Tool Co., Ltd., Ueda, Nagano, Japan) facing the headwind every 30 min. Direct and diffuse solar radiation in the horizontal plane was recorded using a pyranometer (MS-01; Eko Instruments Co., Ltd., Tokyo, Japan) every 30 min, and solar radiation (global) was estimated by summing up the values.

### Calculations

The equations of T_sk_, mean radiant temperature (T_r_), dry or sensible heat loss (DHL), evaporative heat loss (EHL), total heat loss (THL), absolute humidity, total sweat loss and age-predicted maximal HR (HRmax) are included in supporting information (S1 Data).

### Statistical analyses

Data are presented as mean±SD. The significance level was set at *P*<0.05. The normality of the data and the homogeneity of variance between the trials were tested using Shapiro-Wilk’s test and Levene’s test, respectively. Non-parametric data (TS) were analysed using R (version 4.0.2). TS was analysed using a two-way (time-of-day [two levels, i.e., AM and PM] × time [four levels, i.e., 0, 60, 120 and 150 min]) repeated measures ANOVA with the R package nparLD (version 2.1) for the LD-F2 design. Pair-wise differences between trials were evaluated using the Tukey multiple comparison tests. In all other cases, statistical analyses of data were done in the IBM SPSS (version 21; IBM Corp., Armonk, N.Y., USA). Data collected once per trial were analysed using a one-way repeated measures ANOVA, and data collected over time were analysed using a two-way (time-of-day [two levels, i.e., AM and PM] × time [three or four levels, i.e., 0, 60, 120 and 150 min]) repeated measures ANOVA. Pair-wise differences between trials were evaluated using one-way ANOVAs with a Bonferroni adjustment applied for multiple comparisons. Environmental parameters were analysed using the independent (AM vs. PM) and dependent (indoor AM vs. outdoor AM; indoor PM vs. outdoor PM) samples t-test. Cohen’s d (*d*) was used as a measure of effect size for parametric paired samples; a *d* of 0.2 to <0.5 and ≥0.5 to <0.8 has been suggested to represent a small and medium treatment effect, respectively, while a *d* ≥0.8 represents a large treatment effect [18]. Spearman’s rank correlation coefficient (*r*_s_) was used to assess the relationship between the changes in T_ty_, T_sk_ and HR in each subject and the changes in T_a_, WBGT, T_g_, T_r_, and solar radiation at 60, 120 and 150 min. A *r*_s_ of <0.2 were considered a weak correlation, 0.21–0.4 were considered fair, 0.41–0.6 were regarded as moderate, 0.61–0.8 were deemed strong and 0.81–1.0 very strong [19].

## Results

**Fig 2:**
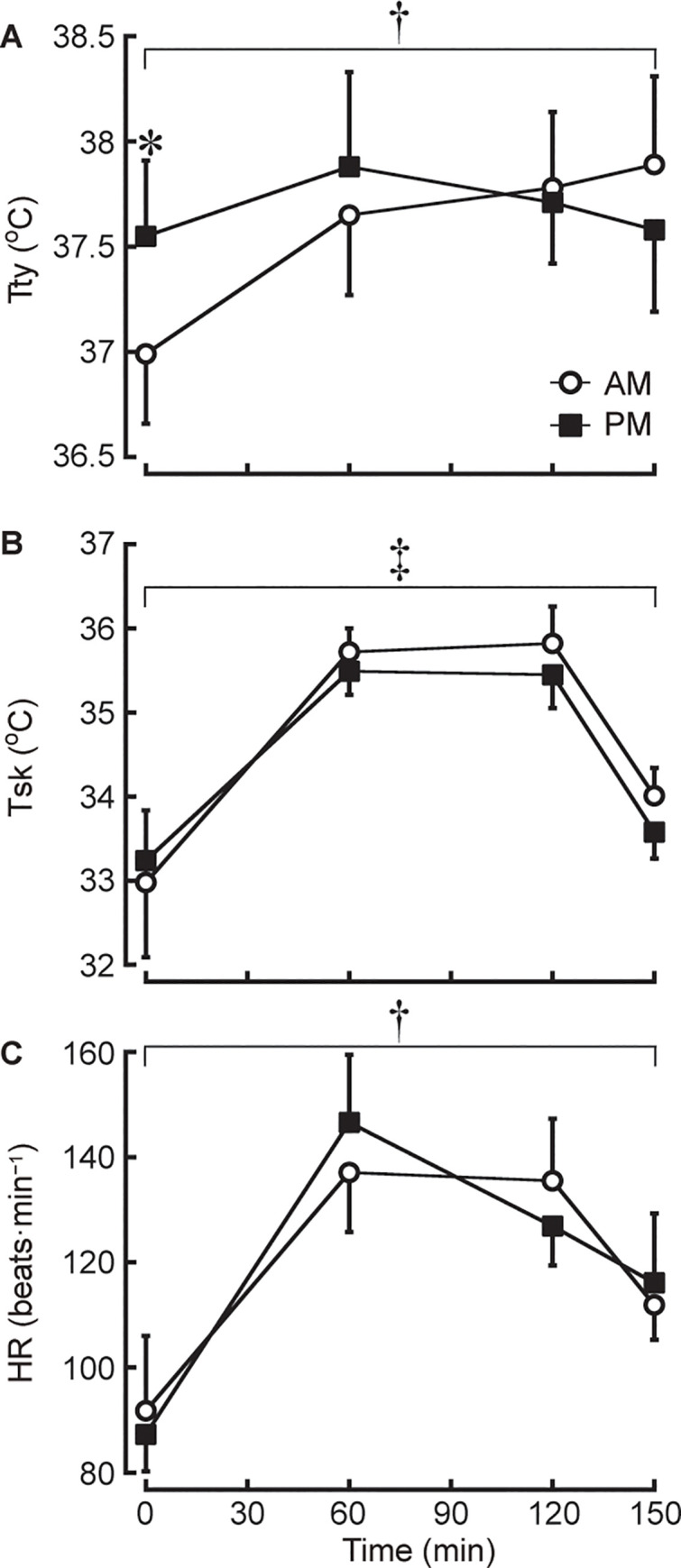
Changes in infrared tympanic temperature (Tty; A), mean skin temperature (Tsk; B) and heart rate (HR; C) during exercise. *P<0.05 denotes a difference of pre-exercise between AM and PM trials. †*P*<0.05 denotes an interaction between AM and PM trials. ‡ *P*<0.05 denotes a main effect of trial between AM and PM trials.

Pre-exercise body mass (*P* = 0.580), T_sk_ (*P* = 0.349) and HR (*P* = 0.247) were not different between trials, but pre-exercise T_ty_ was higher on PM than AM trial (*P*<0.001; 1−β = 1.00; *d* = 1.51; Fig 2A).

### Environmental conditions

In indoor environmental conditions, T_a_ (1−β = 0.97; *d* = 2.23) and T_g_ (1−β = 0.88; *d* = 1.74) were lower and RH (1−β = 0.66; *d* = 1.27) was higher on AM than PM trial (Table 1). In outdoor environmental conditions, absolute humidity (1−β = 0.96; *d* = 2.15) and solar radiation (1−β = 1.00; *d* = 3.19) were higher on AM than PM trial (Table 1).

**Table 1:** Indoor (Judo facility) and outdoor environmental conditions during each trial.

			Time				Mean ± SD	*p* value
	0	30	60	90	120	150		
Indoor environment								
Ta, °C								
AM	32.0	32.4	32.8	33.0	33.0	33.1	32.7 ± 0.4	0.008
PM	35.5	35.3	34.8	34.1	34.0	32.4	34.4 ± 1.0	
RH, %								
AM	64	62	61	62	61	59	61 ± 2	0.037
PM	60	52	52	58	56	62	57 ± 4	
AH, g·m^−3^								
AM	21.7	21.4	21.5	22.1	21.6	21.2	21.6 ± 0.3	0.857
PM	24.4	20.9	20.4	21.9	21.1	21.4	21.7 ± 1.3	
AV, km·h^−1^								
AM	2.5	2.5	2.5	2.5	2.5	2.5	2.5 ± 0.0	1.000
PM	2.5	2.5	2.5	2.5	2.5	2.5	2.5 ± 0.0	
WBGT, °C								
AM	28.8	28.9	29.0	29.3	29.1	29.4	29.1 ± 0.2	0.074
PM	31.6	30.1	29.7	30.0	29.5	28.8	29.9 ± 0.8	
Tg, °C								
AM	34.5	35.0	35.0	34.9	35.1	36.6	35.2 ± 0.7	0.021
PM	38.3	37.5	37.2	36.8	36.4	34.6	36.8 ± 1.1	
Tr, °C								
AM	38.7	39.5	38.9	38.0	38.6	42.4	39.4 ± 1.4	0.114
PM	42.8	41.4	41.2	41.4	40.4	38.2	40.9 ± 1.4	
Outdoor environment								
Ta, °C								
AM	31.9	31.9	32.6	32.9	33.0	33.6	32.7 ± 0.6	0.119
PM	33.5	32.9	31.5	31.0	30.7	30.2	31.6 ± 1.2	
RH, %								
AM	58	58	57	58	58	60	58.2 ± 0.9	0.416
PM	53	55	58	58	59	60	57.2 ± 2.4	
AH, g·m^−3^								
AM	19.5	19.5	19.9	20.6	20.7	22.1	20.4 ± 0.9	0.008
PM	19.4	19.5	19.1	18.6	18.6	18.4	18.9 ± 0.4	
AV, km·h^−1^								
AM	3.0	5.0	5.0	2.0	10.0	7.0	5.3 ± 2.6	0.469
PM	7.0	6.0	4.5	4.0	2.0	2.0	4.3 ± 1.9	
WBGT, °C								
AM	29.0	29.3	29.8	30.2	30.3	31.2	30.0 ± 0.7	0.147
PM	31.0	30.3	28.6	28.2	28.4	27.7	29.0 ± 1.2	
Tg, °C								
AM	42.0	42.5	43.0	43.5	43.8	44.8	43.3 ± 0.9	0.058
PM	45.0	43.2	38.5	38.1	37.2	35.5	39.6 ± 3.4	
Tr, °C								
AM	58.7	65.5	65.6	57.3	77.3	73.2	66.3 ± 7.2	0.124
PM	74.1	67.8	53.9	52.7	46.4	43.1	56.3 ± 11.1	
SR, W·m^−2^								
AM	860	920	930	1020	1060	1100	982 ± 85	<0.001
PM	810	510	300	220	160	110	352 ± 242	

Ta, ambient temperature. RH, relative humidity. AH, absolute himidity. AV, air velocity

WBGT, wet-bulb globe temperature. Tg, black globe temperature. Tr, mean radiant temperature. SR, solar radiation.

In AM trial, higher RH (*P*<0.05; 1−β = 0.90; *d* = 1.81) and absolute humidity (*P*<0.05; 1−β = 0.89; *d* = 1.79) and lower WBGT (*P*<0.01; 1−β = 0.88; *d* = 1.75), T_g_ (*P*<0.001; 1−β = 1.00; *d* = 10.05) and T_r_ (*P*<0.001; 1−β = 1.00; *d* = 5.19) were apparent on the indoor than outdoor environmental conditions. In PM trial, higher T_a_ (*P*<0.001; 1−β = 0.99; *d* = 2.54), absolute humidity (*P*<0.01; 1−β = 1.00; *d* = 2.91) and WBGT (*P*<0.05; 1−β = 0.41; *d* = 0.88) and lower T_r_ (*P*<0.05; 1−β = 0.93; *d* = 1.95) were observed on the indoor than outdoor environmental conditions.

### Body temperature responses

There was a trial by time interaction effect for T_ty_ (*P*<0.05; 1−β = 0.82), but post hoc analysis revealed no difference at any time point between trials (all *P*>0.05; Fig 2A). Also, no main effect of trial was observed in T_ty_ (*P* = 0.137). Although no interaction (*P* = 0.065) was shown in T_sk_ between trials, there was a main effect of trial in T_sk_ (*P*<0.05; 1−β = 0.53) which was higher on AM than PM trial (*P*<0.05; *d* = 0.22: Fig 2B).

### Heart rate response

A trial by time interaction effect was detected for HR (*P*<0.05; 1−β = 0.91), but with post hoc adjustment there was no difference at any time point between trials (all *P*>0.05; Fig 2C). The percentage of HRmax (% HRmax) at 60, 120 and 150 min was 67±6%, 67±6% and 55±3% in AM trial and 72±7%, 63±4% and 57±7% in PM trial. There was a trial by time interaction effect for % HRmax (*P*<0.05; 1−β = 0.87), but post hoc analysis revealed no difference at any time point between trials (all *P*>0.05). The average HR during exercise was not different between trials (AM 63±4% HRmax, PM 64±5% HRmax; *P* = 0.680).

### Heat loss responses

**Fig 3:**
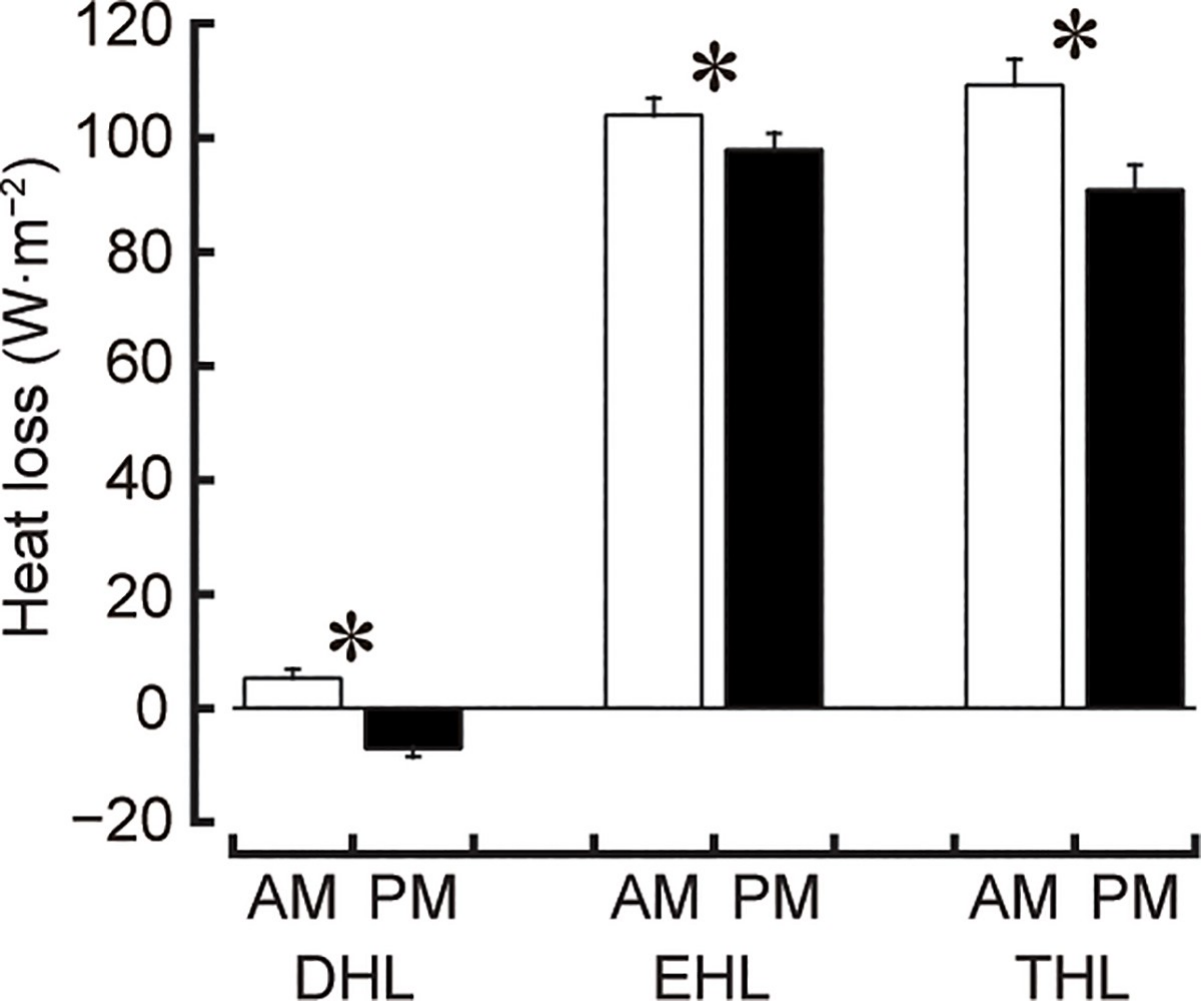
Responses of dry (DHL), evaporative (EHL) and total (THL) heat losses at the skin during exercise. *P<0.001 denotes a difference between AM and PM trials.

DHL (AM 5.3 W·m^−2^, PM −7.0 W·m^−2^; *d* = 7.79), EHL (AM 104.0 W·m^−2^, PM 97.9 W·m^−2^; *d* = 1.92) and THL (AM 109.3 W·m^−2^, PM 90.9 W·m^−2^; *d* = 3.89) were greater on AM than PM trial (all *P*<0.001; all 1−β = 1.00; Fig 3).

### Body fluid balance

There were no differences between trials in the volume of water ingested (AM 2254±718 mL; PM 2269±740 mL: *P* = 0.905), body mass loss (AM 1.2±0.6%; PM 1.3±0.9%: *P* = 0.565), total sweat loss (AM 3.1±0.8 kg; PM 3.1±0.7 kg: *P* = 0.509) and sweat rate (AM 1.23±0.30 L/h; PM 1.26±0.27 L/h: *P* = 0.481).

### Perceptual responses

**Fig 4:**
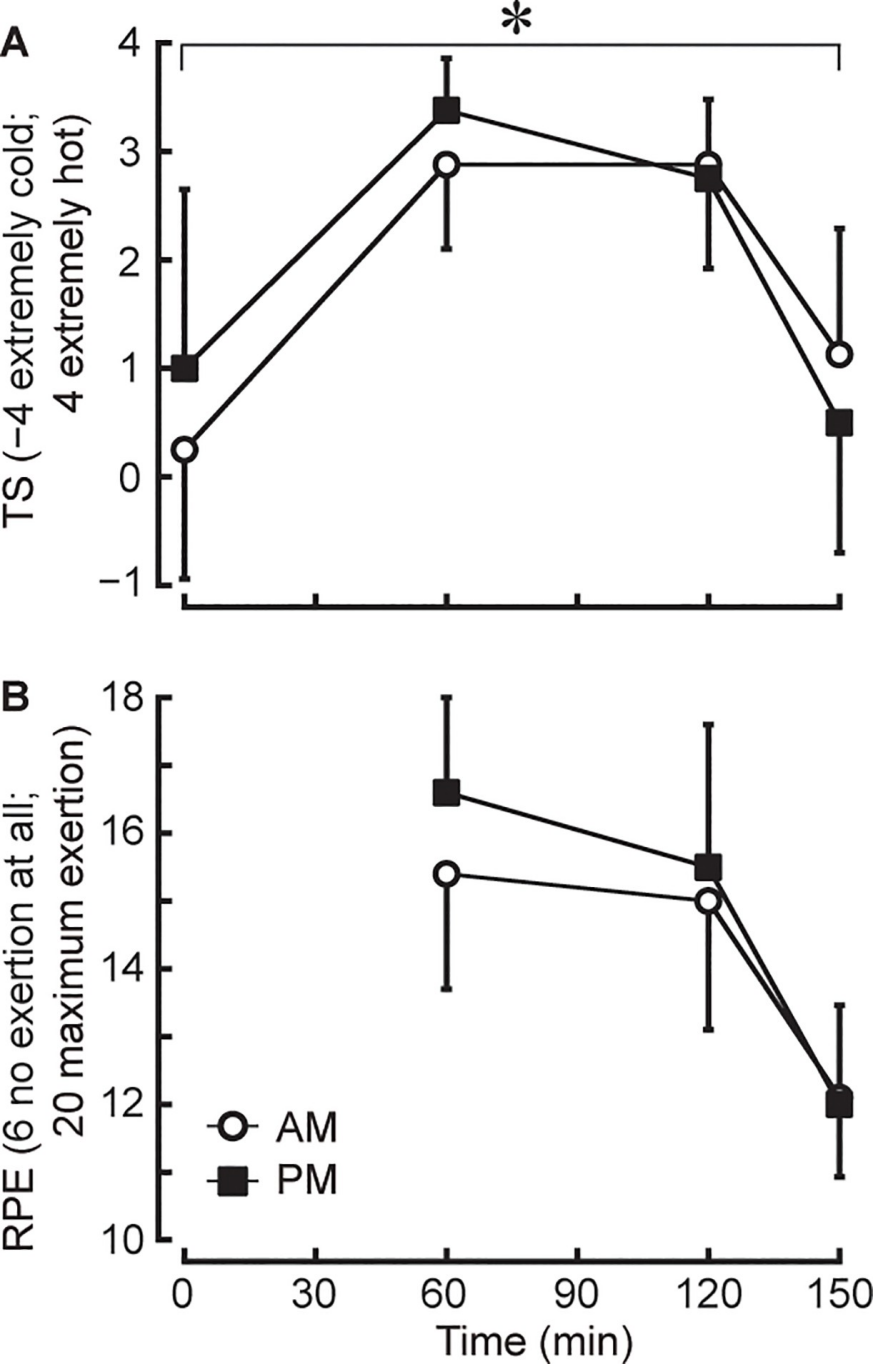
Changes in thermal sensation (TS; A) and rating of perceived exertion (RPE; B) during exercise. *P<0.05 denotes an interaction between AM and PM trials.

A trial by time interaction effect was shown for TS (*P*<0.05), but post hoc analysis revealed no difference at any time point between trials (all *P*>0.05; Fig 4A). Also, there was no main effect of trial in TS (*P* = 0.137). There was no interaction (*P* = 0.214) and main effect of trial (*P* = 0.089) in RPE, although a tendency was observed in a main effect of trial (Fig 4B).

### Relationship between the changes in T_ty_, T_sk_ and HR and in environmental conditions

The changes in Tty was not correlated with the changes in environmental conditions in both trials. The changes in T_sk_ and HR in PM trial was strongly correlated with the changes in T_a_, T_g_, WBGT and T_r_ in the indoor environment and T_g_, WBGT, T_r_ and solar radiation in the outdoor environment (Table 2). Also, the changes in HR in PM trial was moderately correlated with the changes in T_a_ in the outdoor environment.

**Table 2:** Spearman’ s rank correlation coefficianet (*r*_s_) between the changes in mean skin temperature (ΔTsk) and heart rate (ΔHR) in each participant and the changes in indoor and outdoor environmental conditions at 60, 120 and 150 min.

		Indoor					Outdoor		
	ΔTa	ΔWBGT	ΔTg	ΔTr	ΔTa	ΔWBGT	ΔTg	ΔTr	ΔSR
ΔTsk									
AM									
*r*_s_	0.28	0.28	0.28	0.28	0.28	0.24	0.28	0.28	0.28
*p* value	n.s.	n.s.	n.s.	n.s.	n.s.	n.s.	n.s.	n.s.	n.s.
PM									
*r*_s_	0.74	0.74	0.74	0.74	0.08	0.74	0.74	0.74	0.74
*p* value	<0.001	<0.001	<0.001	<0.001	n.s.	<0.001	<0.001	<0.001	<0.001
ΔHR									
AM									
*r*_s_	0.33	0.33	0.33	0.33	0.33	0.28	0.33	0.33	0.33
*p* value	n.s.	n.s.	n.s.	n.s.	n.s.	n.s.	n.s.	n.s.	n.s.
PM									
*r*_s_	0.77	0.77	0.77	0.77	0.52	0.77	0.77	0.77	0.77
*p* value	<0.001	<0.001	<0.001	<0.001	<0.001	<0.001	<0.001	<0.001	<0.001

Ta, ambient temperature. WBGT, wet-bulb globe temperature. Tg, black globe temperature.

Tr, mean radiant temperature. SR, solar radiation.

## Discussion

The current results demonstrated a higher T_sk_ in AM than PM trial (Fig 2B), despite greater DHL and EHL in AM than PM trial (Fig 3) with no differences between trials in hydration status. T_ty_ and HR showed a trial by time interaction effect with no differences at any time point between trials (Fig 2A and 2C), indicating that these were relatively higher in PM than AM trial at the early stages of training but in AM than PM trial at the later stages of training. A novel finding in this study is that there is a greater thermoregulatory strain in the morning from 09:00 h than in the late afternoon from 16:00 h during 2.5-h regular judo training in a judo training facility without air conditioning in the heat of summer. This finding is associated with the progressive increase in indoor and outdoor heat stresses in the morning compared with the progressive decrease in indoor and outdoor heat stresses in the late afternoon. Therefore, our experimental hypothesis was confirmed. These findings are consistent with that of Otani and colleagues which involved 3-h moderate-intensity baseball training [1] and 2-h high-intensity football training [2] in high school athletes in the heat outdoors under a clear sky. As concluded in the previous studies [1, 2], the present study indicates that an increase in indoor heat stress during AM trial may cause a greater thermoregulatory strain than a decrease in indoor heat stress during PM trial, regardless of a smaller indoor heat stress in AM than PM trial and no solar radiation effect on both trials. Hence, the current study supports the previous studies [1, 2] and the risk for developing exertional heat-related illness during exercise may be relatively higher in the morning from 09:00 h than in the late afternoon from 16:00 h when 2.5-h regular judo training is performed in a judo training facility without air conditioning on a clear day in the heat of summer.

Regarding the relationships between indoor heat stress and physiological responses in no air conditioning facility during regular judo training in the summer, only one study of Revera-Brawn & Félix-Dávila [7] has reported the changes in hydration status in adolescent judokas during 90 min judo training in the afternoon from 15:30 h in a temperate environment (29.5°C T_a_). Their study [7] showed that body mass loss, sweat rate and the volume of water ingested during the training were 1.9±0.5%, 0.8±0.3 L/h and 257±246 mL, respectively. These results indicate much greater body mass loss and an excessively lower sweat rate compared with the present results. These disagreements may exist due to the low volume of water ingested and an about 3–5°C lower T_a_ in the previous study [7] compared to the current study. Moreover, no information about exercise intensity was reported in the previous study [7]. Besides, only one study [20] reported the changes in T_ty_ during regular judo training whilst wearing a cooling vest could have attenuated a greater increase in T_ty_ during 65 min regular judo training and 10 min post-training recovery. The authors reported that the temperature was 27°C T_a_ at the beginning of training but they did not state the location where the temperature was measured (indoor or outdoor) and a presence of an air conditioner in the facility [20]. Considering that judo is the most popular combat sport in the world as well as Japanese junior high and high schools, more research is required to evaluate the influence of regular judo training in the heat on the risk of exertional heat-related illness.

In the present study, participants commenced exercise in a judo facility at high WBGT of 28.8°C and 31.6°C at 09:00 h (AM) and 16:00 h (PM), respectively (Table 1), which corresponds to an extreme risk category (≥28°C) for exertional heat-related illness [21]. WBGT kept increasing in AM trial and continued decreasing in PM trial, and WBGT was 29.4°C and 28.8°C at the end of exercise in AM (11:30 h) and PM (18:30 h) trials, respectively (Table 1). These indicate that participants were continuously exposed to heat strain that is regarded as extreme risk for exertional heat-related illness in both trials throughout the training. Indoor heat stress of T_a_, T_g_ and T_r_ also continued to increase during AM trial but they decreased continuously during PM trial that indoor heat stress at the early stages of training was greater in PM than AM trial but the stress at the later stages of training was greater in AM than PM trial (Table 1). Consequently, there was a smaller indoor heat stress with significantly lower T_a_ and T_g_ during exercise in AM than PM trial (Table 1). These changes were well linked to the changes in outdoor heat stress because T_a_, WBGT, T_g_ and T_r_ in outdoors also kept increasing during AM trial and decreasing during PM trial. This study therefore clearly indicates that indoor heat stress increases with increasing outdoor heat stress in the morning or decreases with decreasing outdoor heat stress in the afternoon in a judo training facility without air conditioning on a clear day in the heat of summer. These changes led to greater outdoor than indoor heat stress during the morning in AM trial and greater indoor than outdoor heat stress during the late afternoon in PM trial. This observation is consistent with the common findings of the architectural studies about the diurnal relationships between indoor and outdoor heat stress in a building during the summer [22].

In agreement with the previous studies during outdoor exercise [1, 2], the current study detected a greater thermoregulatory strain during indoor exercise in AM trial than in PM trial. Average HRmax during exercise was 63±4% in AM trial and 64±5% in PM trial which are corresponding to moderate-intensity exercise [23] and similar to the study that was conducted in 3-h moderate-intensity baseball training [1] but lower than the study that was conducted in 2-h high-intensity football training [2]. These studies reported higher T_ty_ and HR [1, 2] and a higher T_sk_ [1] in the morning than in the afternoon trial. In this study, T_sk_ was higher but heat-loss responses of both DHL and EHL were greater in AM than PM trial (Figs 2B and 3). This means a greater heat-gain during exercise in AM than PM trial. T_sk_ at 60 and 120 min of exercise was exceeding 35°C during both trials which is consistent with [2] but higher than [1] the previous studies. It has been known that T_sk_ of greater than 35°C can evoke the early onset of fatigue in a hot environment [24, 25]. Although the present study did not measure exercise performance, the high T_sk_ may have caused an early decline in judo performance in both trials. Given that the changes in T_sk_ in PM trial was related to the changes in T_a_, T_g_, WBGT and T_r_ in the indoor environment (Table 2), a decrease in indoor heat stress in the late afternoon would attenuate a greater increase in T_sk_ in PM trial and which is in line with Otani et al. [1]. Hence, although indoor heat stress was less in AM than PM trial, the progressive increase in indoor and outdoor heat stresses in AM trial may have led to a higher T_sk_ in AM trial compared with PM trial.

The studies of Otani et al. [1, 2] observed an interaction with higher T_ty_ and HR at the later stages of training in the morning than the afternoon trial in the heat outdoors under a clear sky, whereas the current study showed only an interaction with no differences at any time point between trials in T_ty_ and HR (Fig 2A and 2C). Given that the current study detected about 3–8°C lower T_g_ and 12–37°C lower T_r_ than the studies of Otani et al. [1, 2], the impact of solar radiation may have caused the differences in the diurnal impact on T_ty_ and HR responses between the previous and current studies. In this study, the changes in indoor heat stress as measured by T_a_, WBGT, T_g_ and T_r_, T_ty_ and HR showed similar responses where the values were higher in PM than AM trial at the early stages of training, whilst higher in AM than PM trial at the later stages of training. Therefore, it is possible that the changes in T_ty_ and HR responses are easily influenced by the changes in indoor heat stress during indoor exercise in no air conditioning facility in the heat. This may be responsible for the disagreements in the time-of-day influence on T_ty_ and HR responses between the past [1, 2] and current studies.

Perceived thermal stress (i.e. TS) was greater in PM than AM trial at the early stages of training and in AM than PM trial at the later stages of training, even though perceived fatigue (i.e. RPE) was not different between trials (Fig 4). Previous studies demonstrated that RPE and TS responses were almost similar between the morning and afternoon trials during outdoor exercise, although T_ty_, T_sk_ and HR were higher in the morning than the afternoon trial [1, 2]. In the present study, RPE response was consistent but TS response was inconsistent with the previous studies [1, 2]. These results indicate that perceived fatigue during exercise in the heat may not be influenced by the time-of-day and location (indoor or outdoor) when the same training is performed. Meanwhile, Schlader & Vargas [26] reported that central thermoreceptor activation (i.e. ΔT_core_) rather than peripheral thermoreceptor activation (i.e. Δskin temperature) may play a role in perceived thermal stress to exercise in a moderate environment. Although the current study was conducted in a hot environment, statistical analyses revealed the similar changes during exercise between TS and T_ty_ rather than T_sk_. Moreover, given that the changes in TS were also similar to the changes in HR, a higher TS response in PM than AM trial at the early stages of training and in AM than PM trial at the later stages of training would be in accordance with the changes in indoor heat stress as the same responses were observed from T_ty_ and HR. Based on these observations, the chronobiological effect on perceived thermal stress during indoor exercise in the heat may be associated with a combination of the changes in T_core_ and the time-of-day variations in indoor heat stress. Since no studies have systematically examined this effect in any sports including judo, further investigations are required.

The present study is not without limitations. This study used T_ty_ to evaluate T_core_. Nevertheless, previous studies reported that rectal temperature relates to [12] or does not relate to [27] T_ty_ during exercise in the heat. Future research therefore should employ rectal temperature to engage a greater validity and reliability in study regarding the risk of exertional heat-related illness during exercise in the heat of summer. Meanwhile, the current study estimated *f*_cl_, *R*_cl_ and *R*_e,cl_ of judo uniform as 1.23, 0.119 W·(m^2^·°C)^−1^ and 0.022 W·(m^2^·kPa)^−1^, respectively, using that of the similar clothing reported. However, we cannot confirm that whether these estimations are within the acceptable difference for true values. This study observed high T_sk_ of greater than 35°C in both trials that might be accompanied by strong heat which could have accumulated inside the judo uniform. To further elucidate the effects of wearing a judo uniform on heat-gain and -loss responses during judo training in the heat, exact clo values for judo ensemble needs to be established. The present study was conducted in a completely sunny condition. This means that outdoor heat stress continued to increase during the morning and decrease during the afternoon as solar elevation angle rises and falls [28]. That would result in a stable increase of indoor heat stress during AM trial and a stable decrease of indoor heat stress during PM trial in a judo training facility without air conditioning. If the present study was conducted under cloudy conditions, thermoregulatory responses could have been unstable because outdoor and indoor heat stress may not have uniformly increased or decreased. Given this assumption, future study needs to perform the same experiments as the present study under thin or thick cloud conditions.

## Conclusions

We conclude that thermoregulatory strain is greater in the morning from 09:00 h than in the late afternoon from 16:00 h in Japanese high school judokas during 2.5-h regular judo training in a judo training facility without air conditioning on a clear day in the heat of summer. This is attributed to a higher T_sk_ relative to greater DHL and EHL in the morning compared with the late afternoon during exercise, although T_ty_, HR and TS at the early stages of training were higher in the late afternoon than the morning but these at the later stages of training were higher in the morning than in the late afternoon. These findings would be owing to a progressive increase in indoor heat stress with increasing outdoor heat stress in the morning compared with a decrease in indoor heat stress with decreasing outdoor heat stress in the late afternoon when it is a clear day. These observations suggest that judo training in a judo training facility without air conditioning on a clear day in the heat of summer may be at a relatively higher risk for developing exertional heat-related illness in the morning from 09:00 h when the starting WBGT is about 29°C compared with the late afternoon from 16:00 h. Therefore, we suggest conducting judo training in the afternoon from 16:00 h to minimise the risk of developing an exertional heat-related illness in the heat on a clear day, even if the starting WBGT is about 31°C. While an exertional heat-related illness is commonly considered as a risk of outdoor activity, athletes and coaches of indoor sports should recognize that activities in indoor facility do not exempt them from heat stress. Proactive heat mitigation strategies applied in other outdoor sports (e.g. adjustment of work to rest ratio, individualized hydration plan) should also be implemented in Judo and other high intensity indoor sports. As WBGT was exceeding 29°C from the end of AM trial (11:30 h) to the start of PM trial (16:00 h), judo training in such environmental conditions should be avoided to eliminate the mortality and morbidity of heat-related illnesses.

## Supporting information

S1 Data (DOCX)

## References

[1] Otani H, Goto T, Goto H, Shirato M. Time-of-day effects of exposure to solar radiation on thermoregulation during outdoor exercise in the heat. *Chronobiol Int*. 2017; 34(9):1224–1238. 10.1080/07420528.2017.1358735 28910548

[2] Otani H, Goto T, Goto H, Hosokawa Y, Shirato M. Solar Radiation Exposure Has Diurnal Effects on Thermoregulatory Responses During High-Intensity Exercise in the Heat Outdoors. *J Strength Cond Res*. 2019; 33(10):2608–2615. 10.1519/JSC.0000000000003260 31361730

[3] Hatori Y. Heat Stroke in Schools. *Japan Medical Association Journal*. 2013; 56(3):179–185.

[4] Jetté M, Sidney K, Blümchen G. Metabolic equivalents (METS) in exercise testing, exercise prescription, and evaluation of functional capacity. *Clin Cardiol*. 1990; 13(8):555–565. 10.1002/clc.4960130809 . 2204507

[5] Callister R, Callister RJ, Staron RS, Fleck SJ, Tesch P, Dudley GA. Physiological characteristics of elite judo athletes. *Int J Sports Med*. 1991; 12(2):196–203. 10.1055/s-2007-1024667 1860744

[6] Casa DJ, DeMartini JK, Bergeron MF, Csillan D, Eichner ER, Lopez RM, et al National Athletic Trainers' Association Position Statement: Exertional Heat Illnesses. *J Athl Train*. 2015; 50(9):986–1000. 10.4085/1062-6050-50.9.07 . 26381473 PMC4639891

[7] Rivera-Brown AM, De Félix-Dávila RA. Hydration status in adolescent judo athletes before and after training in the heat. *Int J Sports Physiol Perform*. 2012; 7(1):39–46. 10.1123/ijspp.7.1.39 . 21941009

[8] McCullough EA, Jones BW, Huck J. A comprehensive data base for estimating clothing insulation. *ASHRAE Trans*. 1985; 92(2):29–47.

[9] Zuo J, McCullough E. Heat transfer characteristics of sports apparel. *J ASTM Int*. 2004; 1(10):1–10.

[10] Ramanathan NL. A new weighting system for mean surface temperature of the human body. *J Appl Physiol*. 1964; 19:531–533. 10.1152/jappl.1964.19.3.531 . 14173555

[11] Hosokawa Y, Adams WM, Stearns RL, Casa DJ. Comparison of gastrointestinal and rectal temperatures during recovery after a warm-weather road race. *J Athl Train*. 2016; 51(5):382–388. 10.4085/1062-6050-51.7.02 . 27186918 PMC5013699

[12] Otani H, Kaya M, Tamaki A, Hosokawa Y, Lee JKW. Solar radiation and the validity of infrared tympanic temperature during exercise in the heat. *Int J Biometeorol*. 2020; 64(1):39–45. 10.1007/s00484-019-01791-1 . 31473810

[13] Hansen RD, Amos D, Leake B. Infrared tympanic temperature as a predictor of rectal temperature in warm and hot conditions. *Aviat Space Environ Med*. 1996; 67(11):1048–1052. . 8908342

[14] ISO. Ergonomics of the thermal environment—Assessment of the influence of the thermal environment using subjective judgement scales. Geneva: ISO, ISO 10551; 1995.

[15] Otani H, Kaya M, Tamaki A, Goto H, Goto T, Shirato M. Diurnal effects of prior heat stress exposure on sprint and endurance exercise capacity in the heat. *Chronobiol Int*. 2018; 35(7):982–995. 10.1080/07420528.2018.1448855 . 29561175

[16] Otani H, Kaya M, Tamaki A, Watson P, Maughan RJ. Air velocity influences thermoregulation and endurance exercise capacity in the heat. *Appl Physiol Nutr Metab*. 2018; 43(2):131–138. 10.1139/apnm-2017-0448 . 28985477

[17] Borg GA. Psychophysical bases of perceived exertion. *Med Sci Sports Exerc*. 1982; 14(5):377–381. . 7154893

[18] Cohen J. Statistical power analysis for the behavioral sciences. 2nd ed. Hillsdale: Lawrence Erlbaum Associates; 1988.

[19] Landis JR, and Koch GG. The measurement of observer agreement for categorical data. *Biometrics*. 1977; 33(1):159–174. . 843571

[20] Carballeira E, Morales J, Fukuda DH, Granada ML, Carratalá-Deval V, López Díaz de Durana A, et al Intermittent Cooling During Judo Training in a Warm/Humid Environment Reduces Autonomic and Hormonal Impact. *J Strength Cond Res*. 2019; 33(8):2241–2250. 10.1519/JSC.0000000000002443 . 29324576

[21] Armstrong LE, Casa DJ, Millard-Stafford M, Moran DS, Pyne SW, Roberts WO. American College of Sports Medicine position stand. Exertional heat illness during training and competition. *Med Sci Sports Exerc*. 2007; 39(3):556–572. 10.1249/MSS.0b013e31802fa199 . 17473783

[22] Givoni B. Passive and low energy cooling of buildings. New York: John Wiley & Sons; 1994 10.1080/00140139408963659

[23] American College of Sports Medicine (ACSM). Position stand: The recommended quantity and quality of exercise for developing and maintaining cardiorespiratory and muscular fitness, and flexibility in adults. *Med Sci Sports Exerc*. 1998; 30(6):975–991. 10.1097/00005768-199806000-00032 . 9624661

[24] Cheuvront SN, Kenefick RW, Montain SJ, Sawka MN. Mechanisms of aerobic performance impairment with heat stress and dehydration. *J Appl Physiol*. 2010; 109(6):1989–1995. 10.1152/japplphysiol.00367.2010 . 20689090

[25] Sawka MN, Cheuvron,t SN, and Kenefick RW. High skin temperature and hypohydration impair aerobic performance. *Exp Physiol*. 2012; 97(3):327–332. 10.1113/expphysiol.2011.061002 . 22143882

[26] Schlader ZJ, Vargas NT. Regulation of Body Temperature by Autonomic and Behavioral Thermoeffectors. *Exerc Sport Sci Rev*. 2019; 47(2):116–126. 10.1249/JES.0000000000000180 . 30632999

[27] Casa DJ, Becker SM, Ganio MS, Brown CM, Yeargin SW, Roti MW, et al Validity of devices that assess body temperature during outdoor exercise in the heat. *J Athl Train*. 2007; 42(3):333–342. . 18059987 PMC1978469

[28] Otani H, Kaya M, Tamaki A, Goto H, Maughan RJ. Exposure to high solar radiation reduces self-regulated exercise intensity in the heat outdoors. *Physiol Behav*. 2019; 199:191–199. 10.1016/j.physbeh.2018.11.029 . 30471385

