# Supplementary material for: Greater thermoregulatory strain in the morning than late afternoon during judo training in the heat of summer
Source: PLoS One. 2020 Dec 1;15(12):e0242916. doi: 10.1371/journal.pone.0242916 (PMC7707556; doi:10.1371/journal.pone.0242916)
Supplement: S1 Data — (DOCX) [file pone.0242916.s001.docx]

**Methods**

*Calculations*

T_sk_ was calculated using the following equation [Ramanathan 1967]:

T_sk_ = 0.3×chest + 0.3×upper arm + 0.2×thigh + 0.2×calf [ºC].

Convective heat transfer coefficient (*h*_c_) was calculated using the following equation [Parsons 2014]:

*h*_c_ = 8.3v^0.6^ [W·(m^2^·K)^−1^]

where, v is the air velocity in m·s^−1^. Radiative heat transfer coefficient (*h*_r_) was calculated using the following equation [Parsons 2014]:

*h*_r_ = 4*εσ*$\frac{Ar}{A_{D}}$[237.2+(*t*_cl_+T_r_)/2]^3^ [W·(m^2^·K)^−1^]

where, *ε* is the area weighted emissivity of the clothing body surface (assumed to be 0.95 [Nielsen et al. 1988]); *σ* is the Stefan-Boltzmann constant, 5.67×10^−8^ in W·(m^2^·K^4^)^−1^; $\frac{A_{r}}{A_{D}}$ is the effective radiative area of the body (assumed to be 0.73 for standing person [Fanger 1967]); *t*_cl_ is the mean temperature of the clothed body in °C (using the iteration method [Parsons 2014]); T_r_ is mean radiant temperature in °C (using the equation below). Mean radiant temperature (T_r_) was calculated using the following equation [ISO 1998]:

T_r_ = [(T_g_+273)^4^+2.5×10^8^×v^0.6^(T_g_−T_a_)]^0.25^−273 [°C].

Combined heat transfer coefficient (*h*) was calculated using the following equation [Parsons 2014]:

*h* = *h*_c_ + *h*_r_ [W·(m^2^·K)^−1^].

Dry or sensible heat loss at the skin (DHL) was calculated using the following equation [Parsons 2014]:

DHL = (T_sk_−*T*_o_)/[*R*_cl_+(1/*f*_cl_*h*)] [W·m^−2^]

where, *T*_o_ is the operative temperature in °C (using the equation below); *R*_cl_ is the thermal resistance of clothing [0.119 W·(m^2^·°C)^−1^ in the current study]; *f*_cl_ is the clothing area factor (1.23 in the current study). The operative temperature (*T*_o_) was calculated using the following equation [Parsons 2014]:

*T*_o_ = (*h*_r_T_r_+*h*_c_T_a_)/(*h*_r_+*h*_c_) [°C].

Evaporative heat transfer coefficient (*h*_e_) was calculated using the Lewis Relation [Parsons 2014]:

*h*_e_ = 16.5*h*_c_ [W·(m^2^·kPa)^−1^].

Evaporative heat loss at the skin (EHL) was calculated using the following equation [Parsons 2014]:

EHL = [*w*(*P*_sk,s_−*P*_a_)]/[*R*_e,cl_+(1/*f*_cl_*h*_e_)] [W·m^−2^]

where, *w* is skin wettedness (assumed to be completely wet of 1.0 for fully acclimated individuals [Parsons 2014]); *P*_sk,s_ is the partial water vapour pressure at the skin in kPa (assumed to be the saturated water vapour pressure [*P*_sa_] at Tsk which was calculated using equation below); *P*_a_ is the water vapour pressure in the ambient air in kPa (using the equations below); *R*_e,cl_ is evaporative heat transfer resistance of the clothing layer [0.022 W·(m^2^·kPa)^−1^ in the current study]. The saturated water vapour pressure (*P*_sa_) was calculated using Antoine’s equation [Parsons 2014]:

*P*_sa_ = 0.1exp[18.956−4030.18/(T+235)] [kPa]

where, T is a temperature. *P*_a_ was calculated using the following equation [Parsons 2014]:

*P*_a_ = *P*_sa_ × RH [kPa]

where RH is the relative humidity in %. Total heat loss (THL) was calculated using the following equation:

THL = DHL + EHL [W·m^−2^].

Absolute humidity was calculated using the following equation [Parsons 2014]:

Absolute humidity = 2.17·*P*_a_/T [kg·m^−3^]

where, T is a temperature (K). Total sweat loss was estimated using the following equation:

Total sweat loss = body mass loss + the volume of water ingested [L].

HRmax was calculated by subtracting the age from 220.

**References**

Ramanathan NL. A new weighting system for mean surface temperature of the human body. *J Appl Physiol*. 1964; 19:531-533. PMID: 14173555.

Parsons, K. Human thermal environments. London: Tayler & Francis; 2014.

Nielsen, B, Kassow, K, and Aschengreen, FE. Heat balane during exercise in the sun. *Eur J Appl Physiol Occup Phyaiol*. 1988; 58(1-2):189-196. PMID: 3203666.

Fanger, PO. Calculation of thermal comfort: Introduction of a basic comfort equation. *ASHRAE Transactions*. 1967;73(Part 2):III.4.1-20.

ISO. Ergonomics of the thermal environment - Instruments for measuring physical quantities. Geneva: ISO, ISO 7726; 1998.
