# Supplementary material for: Greater thermoregulatory strain in the morning than late afternoon during judo training in the heat of summer
Source: PLoS One. 2020 Dec 1;15(12):e0242916. doi: 10.1371/journal.pone.0242916 (PMC7707556; doi:10.1371/journal.pone.0242916)
Supplement: Attachment — Submitted filename: Response to Reviewer#2 R2.docx [file pone.0242916.s003.docx]

Response to Reviewer 2

We are grateful to reviewer for the critical comments and useful suggestions that have helped us to improve our manuscript. As indicated in the responses that follow, we have taken all these comments and suggestions into account in the second revised version of our manuscript.

Comment#1:

Minor grammatical errors still exist throughout the text that should be easy enough to address with a

thorough review.

Response to Comment#1:

In accordance with the reviewer’s comment, this paper has been re-proofread by a native English speaker. The changes are shown in red in the revised version of text.

Comment#2:

Please specify the version of R.

Response to Comment#2:

We agree with this comment. To add the version of R, we have replaced the following sentences, line 203-205,

“Non-parametric data (TS) were analysed using R. TS was analysed using a two-way (time-of-day [two levels, i.e., AM and PM] × time [four levels, i.e., 0, 60, 120 and 150 min]) repeated measures ANOVA with the R package nparLD”

with

“Non-parametric data (TS) were analysed using R (version 4.0.2). TS was analysed using a two-way (time-of-day [two levels, i.e., AM and PM] × time [four levels, i.e., 0, 60, 120 and 150 min]) repeated measures ANOVA with the R package nparLD (version 2.1) for the LD-F2 design.”.

Comment#3:

An operational definition of HRm ax is needed to provide context for the current results.

Response to Comment#3:

We agree with this comment. In relation to this comment, we have changed the following points,

1) line 196-198: Replaced the following sentence

“The equations of T_sk_, mean radiant temperature (T_r_), dry or sensible heat loss (DHL), evaporative heat loss (EHL), total heat loss (THL), absolute humidity and total sweat loss are included in supporting information (S1 Methods-Calculations).”

with

“The equations of T_sk_, mean radiant temperature (T_r_), dry or sensible heat loss (DHL), evaporative heat loss (EHL), total heat loss (THL), absolute humidity, total sweat loss and maximal HR (HRmax) are included in supporting information (S1 Methods-Calculations).”.

2) S1 Methods-Calculations, the bottom of the text: Add the following sentence

“HRmax was calculated by subtracting the age from 220.”

We are afraid that our statistical adviser (Dr. Jos Feys, University of Leuven) has pointed out that the Tukey multiple comparison tests rather than the Kruskal-Wallis test with the Wilcoxon rank sum test are a practical and powerful approach to multiple testing in this TS data. The results of statistical analysis in the TS data were not different the present results when using the Tukey multiple comparison tests. Therefore, we would like to replace the following sentence, line 205-206,

“Pair-wise differences between trials were evaluated using the Kruskal-Wallis test with the Wilcoxon rank sum test.”

with

“Pair-wise differences between trials were evaluated using the Tukey multiple comparison tests.”

We are most grateful for your comments on our manuscript. We trust that the second revised version of our manuscript is suitable for publication.
